# Impact of TRP Channels on Extracellular Matrix Remodeling: Focus on TRPV4 and Collagen

**DOI:** 10.3390/ijms25073566

**Published:** 2024-03-22

**Authors:** Qin Wang, Chenfan Ji, Patricio Smith, Christopher A. McCulloch

**Affiliations:** 1 Faculty of Dentistry, University of Toronto, Toronto, ON M5G 1G6, Canada; q.wang.a@dentistry.utoronto.ca; 2 Schulich School of Medicine & Dentistry, Western University, London, ON N6A 3K7, Canada; 3 Faculty of Medicine, Pontifical Catholic University of Chile, Santiago 8320165, Chile; psmithf@uc.cl

**Keywords:** collagen, fibroblasts, ion channels, calcium signaling, fibrosis, repair

## Abstract

Disturbed remodeling of the extracellular matrix (ECM) is frequently observed in several high-prevalence pathologies that include fibrotic diseases of organs such as the heart, lung, periodontium, liver, and the stiffening of the ECM surrounding invasive cancers. In many of these lesions, matrix remodeling mediated by fibroblasts is dysregulated, in part by alterations to the regulatory and effector systems that synthesize and degrade collagen, and by alterations to the functions of the integrin-based adhesions that normally mediate mechanical remodeling of collagen fibrils. Cell-matrix adhesions containing collagen-binding integrins are enriched with regulatory and effector systems that initiate localized remodeling of pericellular collagen fibrils to maintain ECM homeostasis. A large cadre of regulatory molecules is enriched in cell-matrix adhesions that affect ECM remodeling through synthesis, degradation, and contraction of collagen fibrils. One of these regulatory molecules is Transient Receptor Potential Vanilloid-type 4 (TRPV4), a mechanically sensitive, Ca^2+^-permeable plasma membrane channel that regulates collagen remodeling. The gating of Ca^2+^ across the plasma membrane by TRPV4 and the consequent generation of intracellular Ca^2+^ signals affect several processes that determine the structural and mechanical properties of collagen-rich ECM. These processes include the synthesis of new collagen fibrils, tractional remodeling by contractile forces, and collagenolysis. While the specific mechanisms by which TRPV4 contributes to matrix remodeling are not well-defined, it is known that TRPV4 is activated by mechanical forces transmitted through collagen adhesion receptors. Here, we consider how TRPV4 expression and function contribute to physiological and pathological collagen remodeling and are associated with collagen adhesions. Over the long-term, an improved understanding of how TRPV4 regulates collagen remodeling could pave the way for new approaches to manage fibrotic lesions.

## 1. Why Examine Regulation of ECM Remodeling?

In this review, we consider how critical properties of Transient Receptor Potential (TRP) channels in general, and TRPV4 in particular, affect discrete cellular functions that regulate the remodeling of collagen, the most abundant protein in mammals [1]. TRP channels play important roles in a broad array of physiological processes to maintain health. TRPV4 channels contribute to health, but their dysfunction also contributes to several diseases that manifest with disturbances of ECM remodeling. To contextualize this focus, we first provide background on the composition, structure, and function of the ECM, and rationalize the importance of studying regulatory systems of collagen remodeling and their relationship to ECM homeostasis. We then assess how TRP channels, and TRPV4 in particular, affect the structure and remodeling of collagen in health. Finally, we consider how TRPV4 channel dysfunction or inappropriate expression contribute to disturbances of ECM remodeling in various diseases.

## 2. What Are Connective Tissues?

In humans and other mammals, connective tissues contain cells and non-cellular ECM molecules that collectively affect the global phenotype of specific organs or tissues. The cellular and molecular constituents of connective tissues define their function. For example, soft connective tissues, which comprise the interstitium of many different organs, surround, support, and enable the function of multiple tissues, including organ parenchyma. The ECM of specialized connective tissues plays important mechanical functions, such as linking muscle to bone insertions through tendons [2]. Cartilage, adipose tissue, and intervertebral discs are broad spectrum examples of connective tissues that play critical physiological roles in organ and tissue function and in the maintenance of health. Other specialized forms of connective tissues (e.g., basal laminae) attach epithelial cells to underlying lamina propria, while mineralized connective tissues, such as bone, provide tissue support, transfer muscle-generated force to effect movement, and serve as protected sites for hematopoiesis in the bone marrow.

The ECM is a complex of molecules secreted by cells that are a combination of water, proteins, and various polysaccharides. Tissue-specific variations in the composition, organization, and modifications of the ECM affect the physical properties and functions of connective tissues in discrete organs [3]. In particular, the abundance of various types of collagens, glycoproteins (such as elastin and fibronectin), proteoglycans (e.g., lumican, biglycan, decorin), and glycosaminoglycans (e.g., heparan sulphate) help to determine the biomechanical and biochemical nature of ECMs. In various ECMs, over 300 different proteins have been identified. Collectively, these proteins are described as the “core matrisome” [3]. In addition, there are many ECM-associated proteins (such as enzymes and growth factors) that are not part of the “core matrisome” but that play important roles in modifying matrix proteins. To enable ECM homeostasis and preservation of tissue phenotypes, the structure, organization, and function of all of these ECM components must be integrated and precisely regulated.

## 3. ECM Remodeling Mechanisms

**Figure 1:**
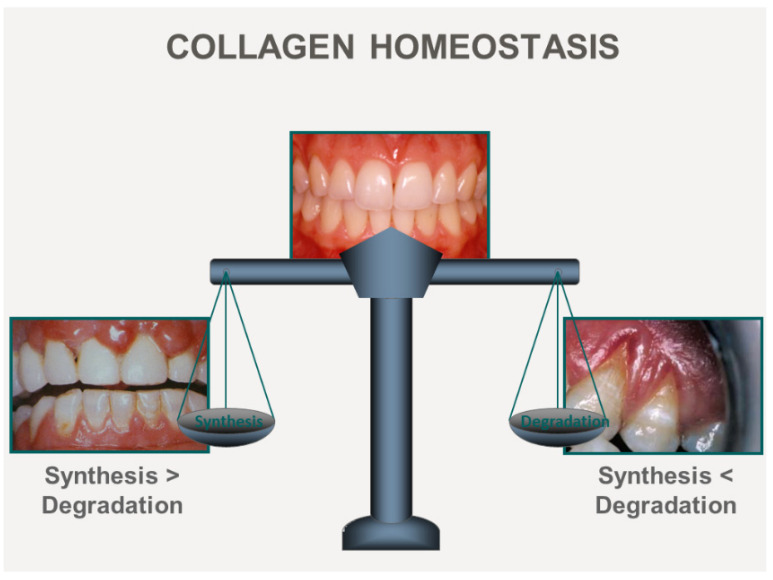
Clinical photographs illustrate the impact of disease on ECM dynamics in periodontal tissues, which constitutively exhibit a rapid rate of collagen turnover. The photograph in the center of the figure shows healthy periodontal tissues, in which synthesis and degradation are in balance. When ECM synthesis exceeds degradation, gingival tissue overgrowth is manifest, as shown in the left panel. When ECM degradation exceeds synthesis, there is net loss of gingival tissue, as shown in the right panel.

The ECM of soft connective tissues, such as the skin dermis and the interstitium of various organs (e.g., kidney, heart and lung, joints, tendon, and periodontal tissues), undergo continuous remodeling by resident fibroblasts to maintain tissue health. Several resident cell types in soft connective tissues actively remodel high abundance matrix proteins, such as fibrillar collagen, so that the normal structure of connective tissues is preserved [4]. For example, connective tissue cells such as fibroblasts and the synoviocytes of joints precisely balance the synthesis, degradation, and reorganization of fibrillar collagen to preserve the structure/function relationships that characterize the ECM of healthy tissues. When the balance between synthesis and degradation of collagen is disturbed because of disease, there are often marked and readily visible disturbances in the structure of collagen-rich tissues (Figure 1).

The cell-mediated processes that enable ECM remodeling rely on a large array of signaling systems. Constitutively expressed, structure-preserving processes that mediate mechanical remodeling of the ECM are dependent in part on intracellular signaling systems. One of these signaling systems involves the precise control of intracellular Ca^2+^ in tissue fibroblasts, which is enabled in part by the expression and function of Ca^2+^-permeable channels. As described below, connective tissue cells can modulate multiple structural, functional, and mechanical properties of the ECM. Cells remodel the ECM by regulating the synthesis, degradation, post-translational enzymatic modifications, and mechanical remodeling of the macromolecular organization of host tissue protein polymers. Remodeling depends in part on Ca^2+^ signaling by resident cells in the local ECM. Ca^2+^-permeable channel expression and dysfunction may underpin the pathogenesis of various types of fibrotic lesions, particularly in the panoply of ECM responses seen in drug-induced lesions of gingiva [5]. Below, we consider how discrete processes in ECM remodeling are affected by Ca^2+^ signaling to rationalize the focus on Ca^2+^-permeable channels.

## 4. Control of ECM Synthesis by Ca^2+^

Interstitial fibroblasts and macrophages are ECM-remodeling cell types that populate the ECM of many soft connective tissues [6]. In certain organs such as the liver or kidney, tissue-specific cells (hepatic stellate cells, renal mesangial cells) can also mediate ECM turnover [7,8]. The ability to define specific cell subpopulations in connective tissues has been facilitated by single-cell RNA sequencing, which show distinct subtypes of fibroblasts based on their localization in tissues, transcriptional profiles, repertoire of expressed molecular markers, and functions [9]. Some of these specialized cell types that are found in connective tissues synthesize and secrete ECM molecules such as collagen fibrils, as well as glycoproteins and proteoglycans [6]. The synthesis of type I procollagen is modified by a large array of regulatory processes. One of these processes involves the release of Ca^2+^ from the endoplasmic reticulum [10] and involves a protein, TRAM2, which is part of the translocon. TRAM2 is needed for collagen synthesis as it couples the activity of SERCA2b with the activity of the translocon. This coupling increases local Ca^2+^ concentration at the site of collagen synthesis, suggesting that high local Ca^2+^ concentrations are needed for the function of molecular chaperones, which in turn are involved in collagen folding prior to export. Ca^2+^ release in human pulmonary fibroblasts responding to ATP (an abundant extracellular nucleotide in inflamed connective tissues) enhances collagen and fibronectin expression [11]. In this process, ATP signals through P2Y receptors to release intracellular Ca^2+^ through ryanodine-insensitive channels, which then mediates the formation of Ca^2+^ waves that increase collagen and fibronectin expression through unknown mechanisms.

## 5. Ca^2+^ Control of Collagen Degradation

As described above, while Ca^2+^ signaling can, under certain conditions, enhance ECM synthesis, Ca^2+^ signaling can also paradoxically promote the breakdown of the ECM. This apparent contradiction is linked to the complementary roles of synthesis and degradation in physiological ECM turnover. In this process, effete matrix molecules are removed and replaced by new ECM to maintain the steady state. The degradation of ECM proteins can be mediated extracellularly by proteolytic enzymes such as matrix metalloproteinases (MMPs) and serine proteases [12]. These enzymes are synthesized and secreted by local connective tissue cells such as fibroblasts, and by inflammatory cells such as neutrophils. These cells express cell-specific types of collagenases (MMP1-fibroblasts; MMP8-neutrophils) that can initiate collagen degradation and thereby enable ECM remodeling. In addition to interstitial collagenases, a large array of soluble MMPs is expressed in connective tissues. The wide substrate specificity of these enzymes [13] enables remodeling of a broad array of different types of ECMs containing diverse ECM molecules.

Earlier data indicated that the concentration of extracellular Ca^2+^ strongly increases the degradation of collagen by interstitial collagenases [14]. Experiments in rabbit synovial fibroblasts employing the Ca^+2^ ionophore, A23187, showed that increased intracellular Ca^2+^ promotes expression of collagenase and stromelysin mRNA, and the secretion of pro forms of these enzymes [15]. Further, the culture of dermal fibroblasts in medium with supranormal Ca^2+^ concentrations enhances the expression and activation of the gelatinases, MMP2 and MMP9 [16]. Conversely, incubation of A2058 human melanoma cells with a calcium influx inhibitor reduced the mRNA expression of MMP1 by >60% [17]. The importance of TRP channels in regulating intracellular Ca^2+^, and therefore ECM degradation, is highlighted by recent data indicating that extracellular digestion of ECM molecules is regulated by TRPV4 through the release and activation of the gelatinases, MMP2 and MMP9 [18].

More localized ECM degradation at specific sites along collagen fibrils is mediated by membrane-associated MMPs, which are tethered to the plasma membrane of fibroblasts and cleave pericellular collagen fibrils. The collagen fragments are subsequently internalized and digested by lysosomal hydrolases such as cathepsins [19]. The degradation of collagen by this intracellular pathway in fibroblasts is an important but poorly understood pathway for the physiological remodeling of mature connective tissues. Phagosomal acidification, which is required for optimization of intracellular collagenolytic activity by cathepsins [20], depends on elevated intracellular Ca^2+^ [20], which suggests a mechanism by which TRP channels, including TRPV4, could control collagen degradation through the internalization pathway.

## 6. Ca^2+^ Control of Post-Translational Modifications of Collagen

In addition to alterations of synthesis and degradation, collagens and elastins undergo post-translational modifications that affect collagen structure and function. Collagen is cross-linked primarily through lysine residues to create large and structurally integrated networks of relatively insoluble proteins, which contribute to the ECM shape and mechanical stability. In tissue development, cross-linking enzymes such as lysyl oxidases and transglutaminases [21] mediate the formation of cross-links, which in turn enhance the tensile strength of matrix polymers and the stiffness of the ECM [22]. These biophysical properties in turn affect cell fate, apoptosis, cell spreading, and the persistence of certain fibroblast subpopulations such as myofibroblasts in fibrotic lesions [23]. In the context of Ca^2+^ signaling and collagen cross-linking, lysyl oxidases contain Ca^2+^ coordination sites that are important for structural stability. But how the function of these enzymes is regulated by Ca^2+^ in general, and by TRPV4 in particular, is not well-defined. TRPV4 is associated with the regulation of certain types of collagen cross-links in megakaryocytes [24], which underlines the importance of this channel in affecting the biophysical properties of the ECM.

## 7. Ca^2+^ Control of Collagen Remodeling by Contraction

One specialized fibroblast subtype, the myofibroblast, is considered to be a well-differentiated cell type that produces abundant ECM, expresses α-smooth muscle actin (α-SMA), and can apply high amplitude contractile forces to matrix polymers, which stiffen and condense collagen fibrils in the ECM. The expression of α-SMA and its incorporation into actomyosin filaments in myofibroblast differentiation is associated with cell contraction [25]. The control of contractile force generation relies in part on intracellular Ca^2+^ concentration and on periodic Ca^2+^ oscillations, which rationalizes the interest in TRP channels as regulators of ECM remodeling mediated by these cells. In myofibroblasts, Ca^2+^ oscillations control contractile activities, while Rho regulates cell tension [26]. As myofibroblasts contribute to wound closure in healing tissue and are also found in fibrotic lesions [27], the role of TRP channels in general, and TRPV4 in particular, is a promising area for future research in the regulation of myofibroblast function and how TRP channels may contribute to fibrosis.

## 8. Polymodal TRP Channels Conduct Ca^2+^ and Regulate ECM Remodeling

As this article is part of a compendium of reviews that considers the structure and function of a variety of TRP channels, we next provide a general background on TRP channels in the context of ECM remodeling. We recently reviewed the classifications, tissue distributions, physiological functions, and structural motifs of TRP channels, and, in particular, the specific structural and functional features of TRPV4 that relate to ECM remodeling [28]. Collectively, the diversity of TRP channel structures is paralleled by a broad array of functions that TRP channels exhibit. These functions are manifest in several different physiological and pathological processes; some impact ECM remodeling in tissue and organ development, as well as in certain diseases in which there is loss of ECM homeostasis (e.g., fibrosis) [29].

TRP channels are expressed by a variety of cell types in multiple organs [28] and are classified as polymodal since they are activated by several different endogenous and exogenous factors. Arising from this breadth of potential activators, TRP channels can sense myriad stimuli including temperature, pain, and taste [30]. Many TRP channels are permeable to Ca^2+^ and can alter intracellular Ca^2+^ concentration to enable signal transduction [31]. In this review, we consider whether dysregulation of Ca^2+^ conductance through TRP channels may contribute to the development and progression of fibrotic lesions, which would expand the role of TRP channels in ECM matrix remodeling. This question is posed in a recent review about the TRP channel, TRPV4 [32]. In addition, TRPM6/7 and TRPC3 channels affect fibroblast proliferation and myofibroblast differentiation in cardiac muscle, and the expression of these channels is increased in cardiac fibrosis [33]. TRPV3 expression and activity are associated with interstitial fibrosis in cardiac tissues, with increased expression of collagens I and III, and enhanced proliferation of fibroblasts [33], while TRPC6 is associated with myofibroblast differentiation in ECM remodeling in the skin, pulmonary, cardiac, intestinal, and renal tissues. There is elevated expression of TRPA1 in fibrotic lesions from patients with Crohn’s disease, and activation of TRPA1 channels in myofibroblasts is associated with reduced intestinal fibrosis after treatment with prednisolone or pirfenidone [34]. In cardiac fibrosis, the expression, pharmacological inhibition, and activation of TRPV1 are associated with depressed fibrotic responses [35], and similarly, reduction of TRPV2 expression is associated with reduced fibrosis in cardiac tissues [36]. In general, the effects of different TRP channels on pro-fibrotic processes appear to be channel-specific, tissue-specific, and disease-specific. Future research will need to consider these multiple factors and the relative importance of the contributions of different TRP channels to health and disease.

## 9. Why Focus on TRPV4?

**Figure 2:**
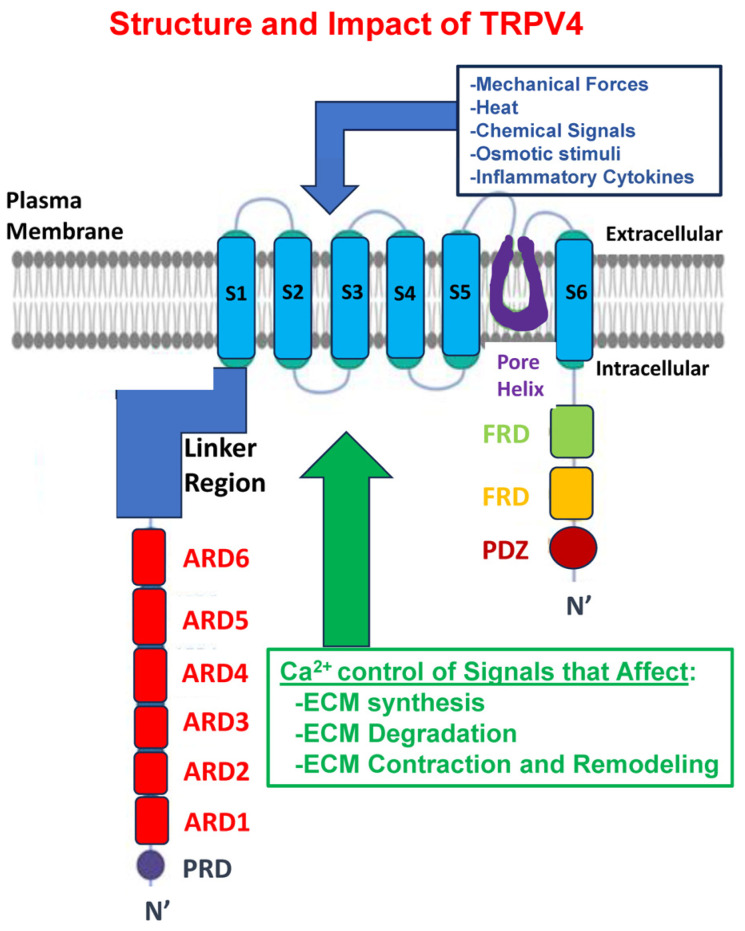
Diagram illustrating the location of TRPV4, embedded in the plasma membrane of connective tissue cells, the various stimuli (in blue) that affect Ca^2+^ conductance, and the cellular impact that TRPV4 exerts on critical elements of ECM homeostatic control.

TRPV4 is one of six members of the TRP-vanilloid subfamily [37,38] and is broadly expressed in different connective tissues and by multiple cell types, including fibroblasts [39] (Figure 2).

Based on current reports describing a relationship between the expression and activity of TRPV4, these channels are of interest in physiological regulation of ECM. A recent review paper has suggested several insightful mechanisms by which TRPV4 mechanotransduction may be involved in the pathogenesis of lung and cardiac fibrosis [32]. This paper helpfully draws attention to the interaction between TRVP4 mechanosensing and some of the other known regulators of fibrosis. Future research examining the phenotypes and lineages of cells that express TRPV4 in fibroblasts, and how specific tissues promote activation of this channel, could provide new insights into fibroblast subpopulations based on TRPV4 expression and activity. Such insights could illuminate how these cells contribute to fibrosis and dysfunctional ECM remodeling.

## 10. Regulation of TRVP4 Expression and Activity

Tissue- and cell-specific expression of TRPV4 and its activity are under tight local control in health, but how these factors contribute to regulation of ECM remodeling, and potentially to matrix dysregulation in disease, is not well-understood. Early studies showed that TRPV4 expression is strongly expressed in the brain and kidney, while more recent work in matrix biology has shown that it is also expressed by fibroblasts and other types of cells in the ECM [28]. Below, we consider how recent advances in TRPV4 regulation may clarify its impact on ECM remodeling.

TRPV4 affects the assembly and turnover of microtubules and actin filaments; conversely, microtubule dynamics regulate TRPV4 activity [40], indicating the existence of a two-way signaling system in which the channel affects the cytoskeleton and vice-versa. The enrichment of TRPV4, actin binding proteins, and actin filaments in the matrix adhesions of fibroblasts may provide an integrated system by which TRPV4/cytoskeletal protein complexes integrate cell structural elements with signal processing molecules. Further, as TRPV4 activity is affected by phosphorylation [41], the enrichment of matrix adhesions with regulatory kinases provides anchorage-dependent cells with localized signaling modules that integrate the ion channel-gating activity of TRPV4 with the ability of cells to “mechanosense” ECM polymers.

The integration of cytoskeletal structures with TRPV4 channel activity is highlighted by recent data that examined how TRPV4 is regulated by RhoA, a small GTPase that affects the assembly of actin stress fibers in cells. The authors found that TRPV4 activation is associated with rotation of the ankyrin repeat domain [42]. The interaction of TRPV4 with membrane-attached RhoA restricts this rotation to regulate channel activity. This mechanism could explain TRPV4′s role in neuromuscular diseases and the actin filament-rich matrix adhesions in fibroblasts that enable the sensing of ECM molecules at cell adhesions.

Since TRPV4 can be activated by mechanical forces including cyclic strain, shear stress, and variations of ECM stiffness, TRPV4 may play a role in fibroblast differentiation and in the pathogenesis of fibrosis, as recently considered [32]. Studies of mechanosensation in endothelial cells indicated that, in response to cyclic stretch, TRPV4 enables Ca^2+^ entry, which is needed for the redirection of cells in response to stretch [43]. Application of tensile and rotational forces to endothelial cells mediates rapid Ca^2+^ transients through TRPV4, which is enriched in focal adhesions containing β1 integrins [44]. The same group showed that TRPV4 expression levels affected the ability of endothelial cells to sense the stiffness of the ECM, and that this sensitivity was in turn affected by the activity of Rho [45]. Taken together, and at least in endothelial cells, the activity and expression levels of TRPV4 are important determinants of mechanosensation.

## 11. TRPV4 Expression and Function Are Associated with ECM Remodeling

Arising from the earlier work in endothelial cells, studies of TRPV4 expression and activity indicate that in fibroblasts, these factors may be important for the processing and integration of mechanosensory signals from the ECM. Several reports indicate that TRPV4 impacts ECM remodeling through its expression levels and/or Ca^2+^ conductance. Increased TRPV4 expression is associated with skin fibrosis in scleroderma [46], while TRPV4 channel activity is enhanced in lung fibroblasts from patients with idiopathic pulmonary fibrosis [47]. On the other hand, pharmacological inhibition of TRPV4 suppresses ECM synthesis in mechanically stressed articular chondrocytes, which are the cells that synthesize and maintain the ECM of articular cartilage. Activation of TRPV4 increases ECM anabolism by chondrocytes [48]. These studies demonstrate potentially important roles of TRPV4 expression and activity in regulating the ECM in tissues with widely varying mechanical properties. As the preservation of tissue-specific mechanical properties of connective tissues is critical for function, there is great interest in how fibroblasts use TRPV4 to sense tissue mechanics and maintain ECM homeostasis. It will be important to discover to what degree TRPV4 (compared with other Ca^2+^-permeable channels) contributes to dysregulated Ca^2+^ signaling and how much of the dysregulation is attributable to alterations of TRPV4 activity and/or expression levels.

## 12. TRPV4 Is Sensitive to Tissue Mechanics in Diverse Processes

One interesting example of TRPV4 sensation of the mechanical properties of the ECM is the observation that TRPV4 expression in mouse skin mediates keratinocyte and fibroblast migration and increases collagen deposition in mouse wounds, thereby promoting cutaneous wound healing through the creation of a stiffer substrate to enhance cell motility [49]. Second, a recent report indicated that TRPV4 was required for the foreign body response involving detection of implant stiffness [50], which was explained as a reciprocal, functional interaction involving TRPV4 sensing and the degree of substrate stiffness. The interaction between TRPV4 sensing and substrate stiffness in turn led to cytoskeletal remodeling and increased force generation by cells. Together, these processes promote the formation of foreign body giant cells.

In pulmonary hypertension, alterations of the mechanical properties of lung connective tissues can drive increased vascular resistance. A recent paper showed that TRPV4 channels mediate fibroblast activation and adventitial remodeling that is seen in the ECM in pulmonary hypertension [51]. In pancreatic fibrosis, which is a frequent complication of chronic pancreatitis and a prominent feature of pancreatic cancer, elevated pressure within the pancreas activates the mechanically sensitive Piezo1 channel. This activation in turn increases Ca^2+^ conductance through TRPV4, thereby leading to stellate cell activation and further pressure-induced chronic pancreatitis and fibrosis [52]. While there are common, underlying mechanisms to explain the role of TRPV4 in these various tissues, one possible mechanism was described earlier in mouse fibroblasts. Ca^2+^ influx through TRPV4 channels regulates the interaction of the actin-binding protein flightless I with the contractile protein non-muscle myosin IIA, which in turn enables generation of the cell extensions and the contractile forces that are essential for collagen remodeling by traction [39].

In musculoskeletal tissues, the load-bearing function of cartilage plays a critical role in preservation of health. For example, off-axis mechanical loading of the spine causes intervertebral disc degeneration and lower back pain. In a mouse model, TRPV4 regulated the expression of COX2/PGE2, which in turn promoted cell-mediated loss of physiological function induced by excessive dynamic compression of the disc. The authors suggested that targeted TRPV4 inhibition may provide a new approach to treat patients suffering from intervertebral disc pathologies caused by excessive mechanical stress [53]. In addition, cyclic-tensile-strain-induced expression of the proteoglycans Acan and Prg4 in annulus fibrosus cells was abrogated after pharmacological inhibition of TRPV4. Conversely, treatment of annulus fibrosus cells with a TRPV4 agonist increased expression of these proteoglycans [54]. In a report on chondrocyte regulatory volume decrease, the TRPV4 channel was evidently involved in the ability of chondrocytes to sense substrate stiffness by mediating Ca^2+^ signaling in a stiffness-dependent manner [55]. Collectively, these reports indicate the broad array of tissues in which TRPV4 plays a regulatory role in the ability of cells to sense ECM mechanics.

In primary keratinocytes, in vitro data indicated that the TRPV4-TAZ mechanotransduction-signaling axis senses matrix stiffness and may impact TGF-β1-induced epithelial-mesenchymal transition [56], an important process in the formation of invasive cancers. Further, signals that are “embedded” in microarchitectural features of the ECM are thought to mediate the ability of aligned fibrillar collagen fibrils to regulate downstream cell signals. TRPV4-mediated Ca^2+^ signaling also regulates force generation in the load-bearing focal adhesion protein vinculin in mesenchymal stem cells, indicating that TRPV4 impacts force generation at cell-matrix adhesions, which are important for the assembly of aligned collagen fibrils [57].

## 13. Relationship between Cell Adhesion Receptors, ECM Remodeling, and TRPV4

Connective tissue cells express ECM adhesion receptors that enable cell attachment, which is required for the migration of anchorage-dependent cells and for the remodeling of the ECM that frequently accompanies migration and that is seen in the compaction of the ECM in fibrotic lesions. Adhesion receptors also translate ECM-encoded biochemical and mechanical signals that affect cell metabolism, thereby enabling two-way communication between cells and the ECM.

The major types of ECM receptors expressed by different types of connective tissue cells and fibroblasts in particular include integrins, discoidin domain receptors, cell-surface proteoglycan syndecans, and hyaluronan receptors such as CD44 [58]. In mammals, various α-integrins (n = 18) and *β*-integrins (n = 8) combine to assemble as *αβ* heterodimers (n = 24 different combinations), which mediate binding to well-defined ECM ligands [52]. For example, collagen-binding integrins contain the *β*1 subunit (*α*1*β*1, *α*2*β*1, *α*10*β*1, and *α*11*β*1). The *β*1 integrin can also associate with other α integrins for binding to other ECM molecules, such as fibronectin. The *β*1 integrin plays an important role in remodeling of the ECM because of its contributions to cell adhesion, migration, collagen degradation by phagocytosis, and control of MMP-mediated collagenolysis. Accordingly, the regulation of *β*1 integrin expression by gene transcription, post-transcriptional processes, and post-translational modifications is important for the maintenance of ECM structure and function.

During remodeling of the ECM, cells apply contractile forces to matrix polymers and condense assemblies of collagen fibrils into stiff aggregates. Studies conducted of cultured cells show that, upon integrin binding and activation by ECM ligands, the cytoplasmic domains of integrins participate in the assembly of cell adhesions, which are termed focal adhesions. There is increased interest in the role of focal adhesions and other types of cell adhesions as they directly impact ECM structure, function, and remodeling, and their structure is influenced by TRPV4 expression [59]. Proteins that associate with the cytoplasmic domains of integrins in focal adhesions include actin binding proteins, such as talin, paxillin, non-muscle myosin, filamins and α-actinins, and signaling proteins (e.g., focal adhesion kinase and Src kinases [60]).

The turnover and stability of focal adhesions are influenced by intracellular Ca^2+^ concentration, which affects Ca^2+^-dependent proteases, such as calpain, that can degrade adhesion-associated proteins. Unexpectedly, high levels of TRPV4 expression are associated with reduced *β*1 integrin abundance and adhesion to collagen, reduced cell adhesion area, and reduced alignment and compaction of extracellular fibrillar collagen. This reduction of *β*1 integrin expression, driven by TRPV4, is dependent on the upregulation of microRNAs that target *β*1 integrin mRNA and affect the abundance of *β*1 integrin transcripts [59]. Expression levels of TRPV4 also affect the size and relative numbers of focal adhesions and the organization of actin-binding proteins in cell adhesions [59], indicating a regulatory role for TRPV4 in the function of adhesive organelles and, potentially, in ECM turnover.

## 14. TRPV4 Signaling and the Fibrotic ECM

In a wide variety of diseases that manifest with chronic inflammation, or after repeated tissue injury, or as a result of certain drugs that affect Ca^2+^ signaling, the regulatory systems that normally preserve ECM tissue homeostasis are disrupted. These disturbances lead to loss of matrix structure and, as a result, disrupted tissue function. In certain instances, disturbed ECM homeostasis arises from impaired Ca^2+^ signaling systems that control the synthesis, degradation, and reorganization of abundant matrix components such as collagen. Examination of animal models shows that TRPV4 knockout mice are shielded from fibrosis in the lung, skin, heart, cornea, and are spared the negative consequences of remodeling in the respiratory tract and the adventitia of blood vessels (review [28]). To add to the complexity of the system, reverse effects are seen in the mouse kidney, as deletion of TRPV4 is associated with more severe renal damage and fibrosis. Part of this latter result may arise from the ability of TRPV4 to enhance myofibroblast differentiation through its transduction of regulatory mechanical signals.

In reactive fibrotic lesions surrounding invasive cancers, some of the resident cell types show high expression levels of the fibrillar collagen-binding, discoidin domain receptor 1 (DDR1), which is linked to increased collagen compaction driven by the association of DDR1 with the Ca^2+^-dependent non-muscle myosin IIA (NMIIA) [61]. In cell culture studies using cells in which the β1 integrin was deleted, compared with DDR1 wild-type cells, high DDR1 expression was linked to increased Ca^2+^ influx through TRPV4. Adjacent to the DDR1-collagen adhesions, TRPV4 was enriched and there was increased non-muscle myosin IIA-mediated contractile activity. At these same cell adhesion sites, there was increased DDR1-mediated collagen alignment and compaction. These data indicate that DDR1 regulates Ca^2+^ influx through the TRPV4 channel to promote DDR1-mediated processes that contribute to the disorganization of collagen seen in fibrotic lesions. Further, in addition to their effects on connective tissue cells, the ECM also underlies the epithelial layer and can therefore affect the behavior of epithelial cells. Studies of collagen matrices with distinct stiffness and microarchitectures showed that the activation of DDR1 is pro-apoptotic and is synergistic with a TRPV4-mediated response to mechanical confinement. The authors suggested that the preference of rhabdomyosarcoma cells to metastasize into microenvironments with low fibrillar collagen concentration, such as the lung, relies on the synergy between TRPV4 and DDR1 [62].

**Figure 3:**
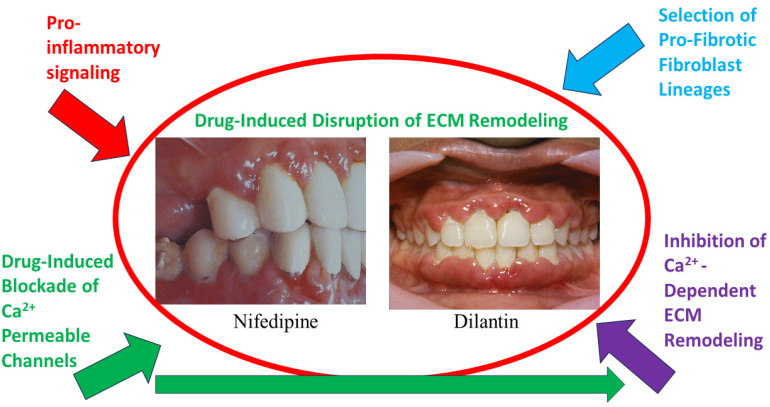
Diagram showing the putative functional relationships between TRPV4, drug-induced blockade of Ca^2+^ conductance, and its effect on ECM remodeling, the simultaneous input from pro-inflammatory signals, the selection for pro-fibrotic fibroblast lineages, and their combined impact on the commonly seen effect of certain drugs (e.g., Dilantin, nifedipine) on gingival overgrowth and disruptions of ECM remodeling.

As described above, when there is a dynamic balance between ECM synthesis and degradation, a steady state is achieved, and ECM homeostasis is generally maintained. Dysregulated ECM remodeling can result in pathological changes to matrix structure that, in turn, interfere with the metabolism and functions of stromal and parenchymal cells, which can culminate in organ failures. As described above (Figure 1), the clinical importance of preserving the balance of ECM synthesis and degradation is critical for the structure and function of periodontal tissues (Figure 3). In health, the balance of ECM synthesis and degradation are kept under tight homeostatic control.

Because of the unusually rapid collagen remodeling that is manifest in healthy periodontal tissues [4], relatively small perturbations of the balance between ECM synthesis and degradation become manifest, so that when collagen synthesis is out of balance with degradation, gingival tissue overgrowth arises. In this context, several drugs that inhibit Ca^2+^ channel function, including cyclosporin [63], Dilantin [64] (Figure 3), and Ca^2+^ channel blockers such as nifedipine (used in the treatment of cardiovascular diseases and hypertension), can promote pronounced gingival overgrowth. While it is not known how these drugs exert such profound, tissue-targeted effects on tissue architecture, there is an apparent dysregulation of Ca^2+^ signaling and possibly Ca^2+^ channels in gingival ECM remodeling in these disorders. Analysis of ECM accumulation, inflammatory infiltrate, and molecular markers in biopsies of drug-induced gingival overgrowth lesions associated with phenytoin, nifedipine, and cyclosporin show considerable heterogeneity, suggesting that these drugs may have different mechanism of action on extracellular matrix remodeling [65]. The effect of cyclosporin A on gingival overgrowth is associated with alterations in intracellular Ca^2+^ signaling [66], which affect intracellular degradation of collagen in lysosomes and then promote the accumulation of collagen in gingival tissues. The recent identification of TRPV4 channels in human gingival fibroblasts and the impact of these channels on collagen remodeling [59] suggest the possibility that some of these gingival overgrowth-promoting drugs may affect TRPV4 function.

## 15. TRPV4 Mutations Manifest as Connective Tissue Abnormalities in Human Disease

Transcriptomic data indicate that TRPV4 expression levels are abundant in the mouse kidney, brain, and bladder, and in the human kidney and salivary gland [67], while there are lower levels in organs of the digestive and nervous systems, heart, lungs, and fat in both mouse and human [68]. These variations suggest that there is tissue-specific TRPV4 availability. Data from analysis of TRPV4 channelopathies show abnormal ECM organization in certain affected tissues. TRPV4 mutations cause groups of diseases that include skeletal dysplasias, peripheral neuropathies, and osteoarthropathy [69], and are predominantly mis-sense mutations with specific amino acid substitutions appearing throughout the channel protein. Patients with certain TRPV4-mediated neuropathies may develop skeletal dysplasias [69,70], which are autosomal dominant disorders that include autosomal dominant brachyolmia type 3, spondylo-epimetaphyseal dysplasia maroteaux pseudo-Morquio type 2, spondylometaphyseal dysplasia Kozlowski type, parastremmatic dysplasia, and metatropic dysplasia. Patients with these disorders exhibit skeletal deformation because of abnormal bone and cartilage growth; they also have shorter trunks and statures. Gain-of-function mutations linked to brachyolmia are associated with increased osteoclast differentiation and bone loss [71]. On the other hand, TRPV4 knockout inhibits osteoclast differentiation and bone resorption, which leads to increased bone mass [72].

Patients with familial digital arthropathy brachydactyly, a type of TRPV4-mediated autosomal dominant arthropathy, exhibit progressive osteoarthritis in the hand and foot joints [69]. In this disorder, the mutations lead to reduced TRPV4 expression and activation in response to stimuli agonists and hypoosmotic stimuli [73], indicating loss-of-function at the cellular level. There is also more cartilage damage in experimental osteoarthritis.

## 16. Conclusions: Roles of TRPV4 in ECM Remodeling

TRPV4 expression and activation are involved in the remodeling of the ECM by regulating ECM protein synthesis and the expression of MMPs.

Dysregulated TRPV4 expression and activation may contribute to disturbed balances of ECM homeostasis.

TRPV4 is sensitive to mechanical stimuli, and its expression and activation may contribute to ECM turnover in response to mechanical forces.

TRPV4 is involved in cell differentiation and proliferation, which can impact ECM remodeling.

The specific organ and tissue context in which TRPV4 is expressed and activated, the cell type, and the local tissue microenvironment, likely play important roles in determining the impact of TRPV4 on ECM turnover and its role in fibrosis.

## Data Availability

The data that support the findings of this study are available in the published literature that is referenced in the manuscript.
